# *Nothodissotis* (Melastomataceae), a new genus from Atlantic Central Africa, including the new species *N.alenensis* from Equatorial Guinea

**DOI:** 10.3897/phytokeys.118.31572

**Published:** 2019-03-07

**Authors:** Marie Claire Veranso-Libalah, Olivier Lachenaud, Robert Douglas Stone, Gudrun Kadereit

**Affiliations:** 1 Institut für Molekulare Physiologie, Johannes Gutenberg-Universität Mainz, D-55099 Mainz, Germany; 2 Institut für Organismische und Molekulare Evolutionsbiologie, Johannes Gutenberg-Universität Mainz, D-55099 Mainz, Germany; 3 Botanic Garden Meise, Nieuwelaan 38, B-1860 Meise, Belgium; 4 Herbarium et Bibliothèque de Botanique africaine, CP 265, Université Libre de Bruxelles, bd du Triomphe, B-1050 Bruxelles, Belgium; 5 School of Life Sciences, University of KwaZulu-Natal, Private Bag X01, Pietermaritzburg 3209, South Africa

**Keywords:** Africa, morphology, *
Dissotis
*, Equatorial Guinea, Melastomataceae, new species, *
Nothodissotis
*, phylogeny, plant conservation, vulnerable species

## Abstract

Based on morphological and phylogenetic evidence, a new genus of Melastomataceae (Melastomateae), *Nothodissotis* Veranso-Libalah & G.Kadereit, **gen. nov.**, is described from Atlantic Central Africa. *Nothodissotis* is distinguished from other African Melastomateae genera by its calyx-lobes that are notched at apex and asymmetrical (vs. entire and symmetrical). *Nothodissotis* includes two species: the type species *N.barteri* (Hook.f.) Veranso-Libalah & G.Kadereit, **comb. nov.** (syn. *Dissotisbarteri* Hook.f.), and the new species *N.alenensis* Veranso-Libalah & O. Lachenaud, **sp. nov.**, described and illustrated here. Both species are restricted to open vegetation on rock outcrops within the forested region of Atlantic Central Africa. *Nothodissotisbarteri* has a scattered distribution in Cameroon, Equatorial Guinea, Gabon and Príncipe Island, while *N.alenensis* is endemic to the Monte Alén massif in Equatorial Guinea, an area where *N.barteri* does not occur. *Nothodissotisalenensis* differs from *N.barteri* by its hypanthium bearing sessile appendages with penicillate hairs (vs. stalked stellate appendages) and its staminal appendages that are much smaller in antepetalous than in antesepalous stamens (vs. subequal in all stamens). The conservation status of both *N.barteri* and *N.alenensis* is assessed as Vulnerable in accordance with IUCN criteria.

## Introduction

Melastomataceae are a large pantropical family with about 4700 species in 170 genera (Clausing and Renner 2001). The majority of their species (c. 3000) occur in the Neotropics, with an important secondary centre of diversity in tropical Asia (c. 1000 species). Continental Africa is relatively poor with c. 330 species, while Madagascar has about the same number (Renner 1993). Most African representatives of the family belong to the pantropical tribe Melastomateae (excluding *Marcetia* DC. and allies now treated in Marcetieae), which includes about 650 species in 32 genera (Michelangeli et al. 2013; Veranso-Libalah et al. 2017a; Rocha et al. 2018). In continental Africa, around 186 species in 13 genera of Melastomateae are currently recognised (Veranso-Libalah et al. 2017a, b). *Dissotis* Benth. has long been regarded as the largest African genus of the tribe, with about 120 species on the continent (Renner 1993) and a single species in Madagascar (Jacques-Félix 1995). Its delimitation, however, has been problematic (Fernandes and Fernandes 1969; Jacques-Félix 1981, 1995), and phylogenetic study has shown the genus to be polyphyletic (Veranso-Libalah et al. 2017a). As a result, the genera *Dissotidendron* (A.Fern. & R.Fern.) Veranso-Libalah & G. Kadereit, with 11 species, and *Dupineta* Raf., with five species, both previously regarded as subgenera of *Dissotis*, have been segregated from the latter. The rest of the *Dissotis* species form a clade together with *Antherotoma* Naudin, *Chaetolepisgentianoides* (Naudin) Jacq.-Fél. (formerly treated in *Nerophila* Naudin) and African species of *Osbeckia* L. (sensu Jacques-Félix 1995), and are paraphyletic with respect to these three genera; the phylogenetic relationships and revised taxonomy of this group (hereafter referred to as ‘*Dissotis* and allies’) are the subject of a forthcoming paper (Veranso-Libalah et al. in prep.).

**Figure 1.**
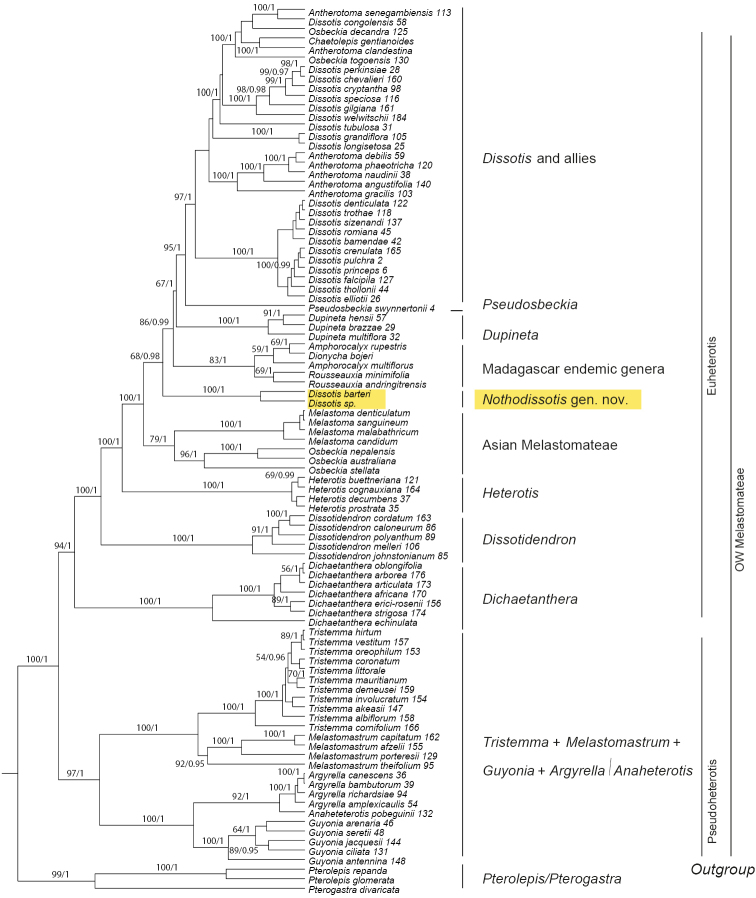
Bayesian maximum clade credibility tree of African Melastomateae based on nuclear (nrITS and nrETS) and plastid (*accD-psaI, ndhF* and *psbK-psbL*) matrices. Values above branches refer to bootstrap values resulting from the ML analysis (only values ≥ 50) and posterior probabilities resulting from Bayesian inference (only values ≥ 0,95). Modified from Veranso-Libalah et al. (2018).

The affinities of the little-known Central African species *Dissotisbarteri* Hook.f. were not investigated by Veranso-Libalah et al. (2017a). However, this species was included in a later phylogenetic and biogeographical study of the group (Veranso-Libalah et al. 2018) using three plastid (*accD-psaI*, *ndhF* and *psbK-psbL*) and two nuclear markers (nrETS and nrITS). In that study, *D.barteri*, together with an undescribed species from Equatorial Guinea, were recovered in a monophyletic clade separate from *Dissotis* and allies (see Fig. 1). Jacques-Félix (1981, 1983a) had previously treated *D.barteri* in D.sect.Macrocarpae A.Fern. & R.Fern., but this is not supported by its morphology or by our molecular phylogenetic results (Veranso-Libalah et al. 2018). Both *D.barteri* and the new species from Equatorial Guinea differ from the members of D.sect.Macrocarpae (and indeed from the rest of the genus) by being deciduous (vs. evergreen) shrubs, and by their calyx lobes that are notched at apex and asymmetrical (vs. entire and symmetrical). Therefore, both molecular and morphological evidence support their exclusion from *Dissotis*.

The above-mentioned new species was previously misidentified as *Dissotisthollonii* Cogn., and was cited under this name in Parmentier and Geerinck’s (2003) checklist of Equatorial Guinean Melastomataceae. These authors reported 16 genera and 57 species of Melastomataceae from Equatorial Guinea, including five *Dissotis* species: *D.barteri*, *D.congolensis* (Cogn.) Jacq.-Fél., *D.hensii* Cogn. [≡ *Dupinetahensii* (Cogn.) Veranso-Libalah & G.Kadereit], *D.multiflora* (Sm.) Triana [≡ *Dupinetamultiflora* (Sm.) Raf.] and *D.thollonii*. While the first four species were correctly identified, *D.thollonii* does not occur in Equatorial Guinea, and most of the specimens cited under this name in the checklist (*Parmentier & Esono 1530, 2721, 2763* and *3453*) actually represent our new species. As discussed above, this species is very close to *D.barteri*, being a ramose shrub with stems and leaves bearing simple hairs, inflorescences few-flowered, and calyx-lobes asymmetrical, while *D.thollonii* is an unbranched shrub with hairs of the vegetative parts more or less branched, inflorescences many-flowered, and calyx-lobes symmetrical. Parmentier and Geerinck (2003) cited two other specimens under *D.thollonii*, *Lejoly 99/004* and *99/345*, of which the former has not been traced (it is apparently not in BRLU), while the latter is sterile and cannot be identified, but differs from the other four collections in vegetative characters.

In this paper we describe a new genus of African Melastomateae, *Nothodissotis* Veranso-Libalah & G.Kadereit, to accommodate both *Dissotisbarteri* and the new species from Equatorial Guinea discussed above. The former species becomes *Nothodissotisbarteri* (Hook.f.) Veranso-Libalah & G.Kadereit, while the latter is described as *N.alenensis* Veranso-Libalah & O.Lachenaud. A review of relevant literature (Keay 1954; Fernandes and Fernandes 1969, 1978; Wickens 1975; Jacques-Félix 1983a, 1983b) confirms that *N.alenensis* differs from all taxa of African Melastomateae so far described.

Material from the following herbaria was consulted for this paper: BR, BRLU, C, EA, K, MO, P, UPS and WAG (Thiers 2018). The description of the new species is based on herbarium specimens and data derived from field notes; all measurements (except plant height) thus refer to dry or rehydrated material were made for both species, following the IUCN criteria (IUCN 2012). The extent of occurrence (EOO) and area of occupancy (AOO) were estimated using GeoCAT (Bachman et al. 2011) with a cell width of 2 km. A distribution map is provided for both species of *Nothodissotis*, as well as a key to the species of the genus, and a key to the currently recognized genera of African Melastomateae.

## Taxonomic treatment

### Nothodissotis

Taxon classificationPlantaeMyrtalesMelastomataceae

Veranso-Libalah & G.Kadereit gen. nov.

urn:lsid:ipni.org:names:60478296-2

#### Type.

*Nothodissotisbarteri* (≡ *Dissotisbarteri* Hook. f.)

#### Morphological diagnosis.

*Nothodissotis* species resemble *Dissotis* by their 5-merous flowers, calyx with caducous lobes and tube not accrescent on the fruit, presence of intersepalar appendages, dimorphic stamens with the connective bearing bipartite ventral appendages and a well-developed pedoconnective, anthers opening by an introrse apical pore, and cochleate seeds. They differ by being deciduous shrubs (vs. evergreen shrubs and herbs) and having the calyx-lobes notched at apex and asymmetrical (vs. entire and symmetrical); the latter character is unique within African Melastomateae.

#### Description.

**Figure 2.**
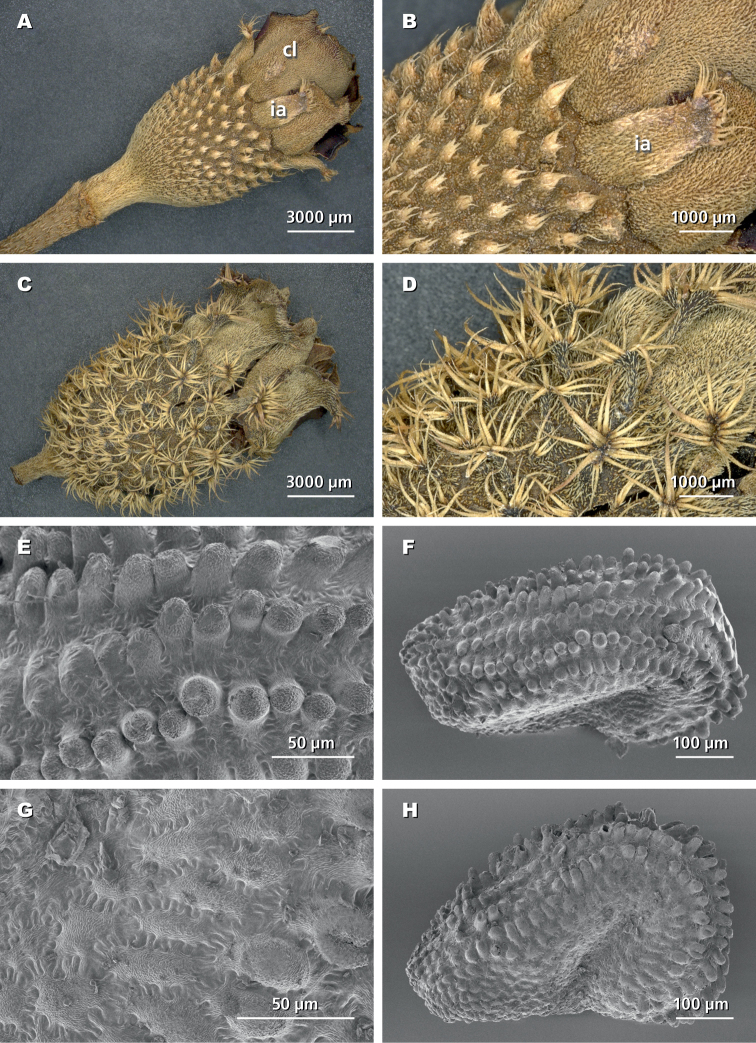
Digital microscope photographs of the hypanthia of *Nothodissotis* spp. (**A–D**) and SEM photographs of the seeds of *N.barteri* (**E–H**). **A, B** hypanthium of *Nothodissotisalenensis* (*Parmentier & Esono 3453*); cl = calyx-lobes and ia = intersepalar appendages **C, D** hypanthium of *N.barteri* (*Ngok Banak 1196*) **E, F** seeds of *N.barteri* in dorsal view **G, H** same in lateral view (*Parmentier 3544*).

**Figure 3.**
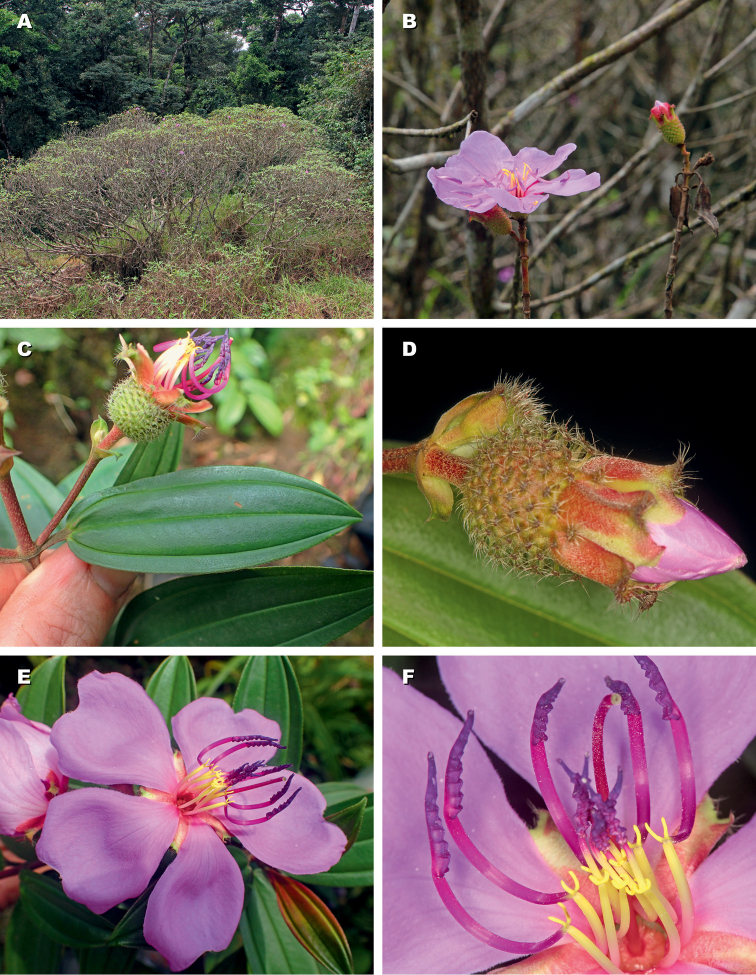
*Nothodissotisbarteri*. **A** habit **B** branches and inflorescence **C** leaf seen from above, and flower (petals fallen) **D** flower bud **E** blooming flower **F** stamens. From *Droissart et al. 1668* (**A, B**) and *Stévart & Oliveira 5136* (**C–F**).

Deciduous, ramose shrubs, 1.5–4 m tall; stems 4-angular to cylindrical, glabrous or strigillose; internodes short, nodes setulose or strigillose (Figs 2, 3). Leaves elliptic with appressed hairs on both sides; 3–5-nerved from the base, margins entire or minutely serrulate. Inflorescences terminal, with 1–7(–15) flowers, flowers 5-merous, pedicellate, subtended by a pair of caducous short ovate bracts. Hypanthium broadly urceolate, with scattered appendages, these either sessile and penicillate (*N.alenensis*; Fig. 2) or stipitate and bearing a stellate crown of hairs at their apex (*N.barteri*; Figs 2, 3). Intersepalar appendages present, similar to hypanthial appendages but much larger, and caducous (Fig. 2 A–D). Calyx-lobes contorted in aestivation and completely concealing the floral buds, elliptic to obovate, asymmetrically notched and bearing penicillate hairs at apex, uniformly pubescent outside, caducous. Petals mauve, broadly obovate, glabrous except for the ciliate margin. Stamens 10, dimorphic in size but not in colour, pedoconnective well-developed, connective with bipartite ventral appendage, anther falcate, opening by an introrse apical pore. Ovary with a crown of persistent bristles, style simple, linear, glabrous. Fruits capsular, enclosed within the hypanthium, splitting loculicidally in 5 valves, the seeds attached on placentas borne on a central column. Seeds (only known in *N.barteri*; see Fig. 2 E–H) cochleate, exarillate, with parallel rows of tubercles.

#### Etymology.

Derived from the Greek word ‘*nothos*’ meaning false, and *Dissotis*, the genus which *Nothodissotis* most closely resembles.

#### Distribution and habitat.

**Figure 4.**
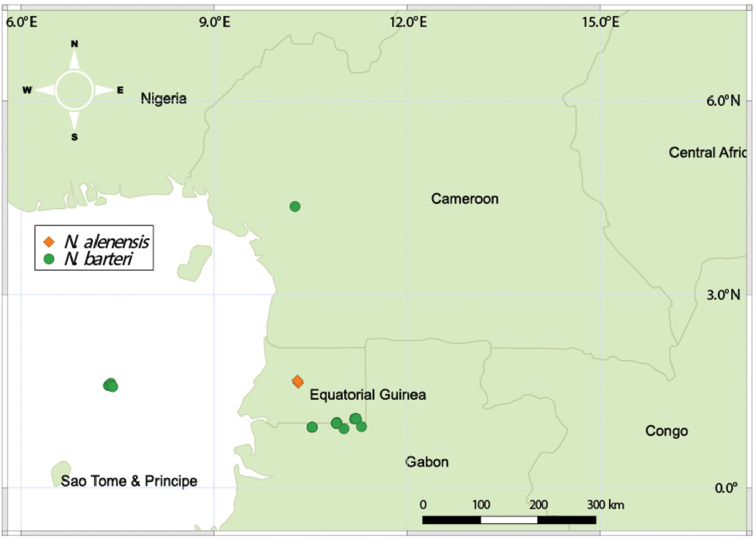
Distribution of *Nothodissotis* species.

*Nothodissotis* includes two species in Atlantic Central Africa, both of which are restricted to rocky outcrops within the equatorial rainforest zone (Fig. 4).

##### Key to the species of *Nothodissotis*

**Table:** 

1	Hypanthium with stalked stellate appendages; staminal appendages of all stamens ± equal in length, but those of antesepalous stamens more distinctly curved; Cameroon, Equatorial Guinea (excluding Monte Alén), Gabon, Príncipe island	*** N. barteri ***
–	Hypanthium with sessile appendages bearing simple penicillate hairs; staminal appendages much smaller in antepetalous than in antesepalous stamens; Equatorial Guinea (Monte Alén)	*** N. alenensis ***

### Nothodissotis barteri

Taxon classificationPlantaeMyrtalesMelastomataceae

(Hook.f.) Veranso-Libalah & G.Kadereit comb. nov.

urn:lsid:ipni.org:names:60478333-2

Figs 2C–H , 3

≡ Dissotisbarteri Hook.f., Fl. Trop. Afr. [Oliver et al.] 2: 454 (1871).

#### Lectotype (designated here).

Príncipe, 1859, *Barter s.n.* (K ! [K000313101]; isolectotype: K ! [K000313102]).

#### Additional specimens examined.

CAMEROON. Réserve de Faune d’Ebo, village de Ndokbaguengue, campement de Djouma, sommet après le transect “Gachaka”, 4°21.7164'N, 10°14.9694'E, 1003 m, 15 Feb. 2014 (fl.), *Droissart et al. 1668* (BRLU!, MO!); Ebo forest proposed National park, Ebo Forest Research Station, Bekango trail, 13 Dec 2006 (fl.), *Osborne & Emang Abwe 323* (K!). EQUATORIAL GUINEA. Inselberg Acoak Banga près de Ngong Mocomo, 1°04'N, 11°11'E, 8 Aug 1998 (fr.), *Lejoly & Elad 98/77* (BRLU!); inselberg de Akoak Ebanga à 1 h du village de Ngong Mocomo, à 10 km de Nsork, 1°04'N, 11°12'E, 585 m, 31 May 2002 (fr.), *Parmentier & Esono 3495* (BRLU!); ibid., 590 m, 1 Jun 2002 (fr.), *Parmentier & Esono 3544* (BRLU!). GABON. rocher Fané, Efout, E of Médouneu, 5 Feb. 1968 (fl.), *N. Hallé & Villiers 4952* (P05264604!); inselberg Milobo, 0°56.35'N, 10°31.31'E, 750 m, 8 Jul 2001 (st.), *Ngok Banak et al. 39* (BRLU!, WAG!); ibid., 0°56.35'N, 10°30.94'E, 760 m, 26 Nov 2001 (fl. buds), *Ngok Banak et al. 301* (BRLU!, WAG!); ibid., 0°56.29'N, 10°30.87'E, 770 m, 6 Dec 2001 (fl.), *Ngok Banak et al. 357* (BRLU!, WAG!); c. 9 km ESE of Médouneu, Efot, inselberg Voma, 1°00.92'N, 10°54.30'E, 500 m, 24 Dec 2002 (fl.), *Ngok Banak et al. 1196* (BRLU!, MO!, WAG!); ibid., 1°00.19'N, 10°54.08'E, 523 m, 26 Dec 2002 (fl. & fr.), *Ngok Banak et al. 1264* (BRLU!, WAG!); Mont Mengong, inselberg au pied du village de Nzec 1, à 45 km de Mitzic vers Sam, 0°57'N, 11°17'E, 670 m, 6 Jan 2000 (fl), *Parmentier & Nguema 585* (BRLU!); Mont Fene, inselberg au pied du village d’Efot, 1°00'N, 10°54'E, 15 Jan 2000 (st.), *Parmentier & Nguema 650* (BRLU!); Mont Voma, inselberg au pied du village d’Efot, 1°00'N, 10°54'E, 19 Jan 2000 (fl.), *Parmentier & Nguema 745* (BRLU!, WAG!); c. 28 km ESE of Médouneu, 0°55'N, 11°01'E, 500 m, 3 Feb 1986 (fl.), *J.M. & B. Reitsma 1796* (WAG!). PRÍNCIPE. Infante D. Henrique, c. 215 m (c. 700 ft), 21 Dec. 1932 (fl.), *Exell 652* (BR0000017285346); sommet du Pico, 30 Aug 1999 (fl.), *Joffroy 202* (BRLU!); Pico Mesa, 600 m, 25 Mar. 1998 (fr.), *Oliveira 546* (BRLU!); Morro Fundao, 1°37'N, 7°23'E, 370 m, 8 Oct. 1997 (fr.), *Stévart & Oliveira 259* (BRLU!). Príncipe Island, summit of the Pico de Príncipe, submontane forest with many epiphytes on a ridge, 01°34'48"N, 007°23'01"E, 945 m, 16 Feb. 2018 (fl. & fr.), *Stévart & Oliveira 5136* (BRLU!, MO!).

#### Distribution and habitat.

*Nothodissotisbarteri* is sparsely distributed in Cameroon (Ebo forest), south-eastern Equatorial Guinea (near Nsork), northern Gabon, and Príncipe Island (Fig. 4). It occurs exclusively on rock outcrops at 370–1000 m elevation, mainly in low shrubby vegetation near the edge of the rocks (“manteau arbustif”) where it is locally dominant, and sometimes also as isolated plants in rocky grassland dominated by *Afrotrilepispilosa* (Boeck.) J.Raynal (Cyperaceae).

#### Phenology.

Flowering recorded mainly from November–February, once in August; fruits in March, May–June, August, October and December.

#### Conservation status.

Vulnerable [VU B2ab(iii)]. The EOO of *Nothodissotisbarteri* is estimated to be 82,625 km^2^ (above the upper limit for Vulnerable status under sub-criterion B1) and its AOO to be 48 km^2^ (within the limit for Endangered under sub-criterion B2). The species is sparsely distributed in Cameroon, Equatorial Guinea, northern Gabon and Príncipe island, and is restricted to rocky outcrops where it occurs in low shrubby vegetation or grassland. It is known from 21 collections representing eleven subpopulations, most of which (except three on Príncipe island) lie outside protected areas. In most of its range, bushfires and agriculture (mostly pineapple plantations) represent the main threats to its habitat; planned tourism development in Príncipe is another threat. A decline in habitat extent and quality is therefore expected. The eleven subpopulations represent a total of ten locations (sensu IUCN 2012), falling within the limit for Vulnerable status, and the species is therefore assessed as Vulnerable under these conditions B2ab(iii).

#### Notes.

This species, originally described from Príncipe Island (Hooker 1871), has since been reported from Gabon (Jacques-Félix 1983b) and Equatorial Guinea (Parmentier and Geerinck 2003). The collections cited above from Cameroon are the first for the country and represent an important range extension northwards.

The seeds of this species have not been described previously (e.g. Jacques-Félix 1983b). They are cochleate, c. 0.5 × 0.35 mm, and bear rounded tubercles arranged in parallel rows (Fig. 2 E–H).

Two specimens, probably from the same field collection by Barter in 1859, are housed in K, with neither of them designated as the holo- or isotype. For this reason, we designate the specimen K000313101 as the lectotype and K000313102 as the isolectotype.

### Nothodissotis alenensis

Taxon classificationPlantaeMyrtalesMelastomataceae

Veranso-Libalah & O. Lachenaud sp. nov.

urn:lsid:ipni.org:names:60478297-2

Figs 2A, B ; 5

#### Type.

EQUATORIAL GUINEA. Río Muni: Monte Alén National Park, Engong rock slab, 5 km west of Engong village, 1°37'N, 10°18'E, 1100 m, 11 May 2002 (fl & buds), *Parmentier & Esono 2763* (holotype: BRLU! [BRLU0000194]; isotype: BRLU! [BRLU0000197]).

#### Diagnosis.

This new species differs from *N.barteri* by its hypanthial appendages that are sessile with penicillate hairs (not stipitate with a crown of stellate hairs) and its more strongly dimorphic stamens, the staminal appendages being much longer in antesepalous stamens than in antepetalous ones (vs. staminal appendages ± equal in length in all stamens).

#### Description.

**Figure 5.**
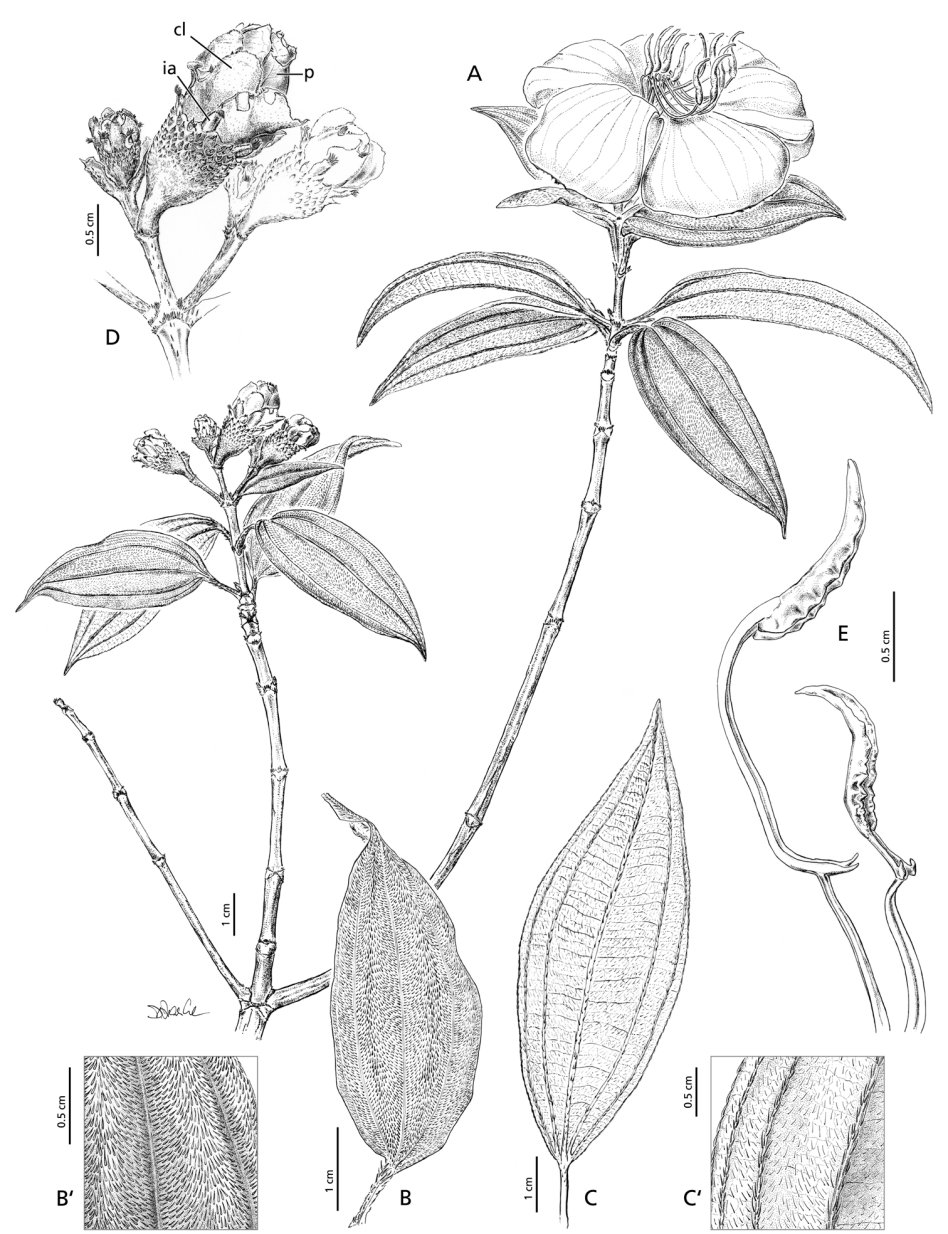
*Nothodissotisalenensis*, **A** habit **B, B**´ leaf adaxial surface **C, C**´ leaf abaxial surface **D** floral buds in different developmental stages; cl = calyx-lobes, ia = intersepalar appendages, p = petals **E** stamens of the outer (left) and inner (right) stamen whorls (drawn from *Parmentier & Esono 1560, 2721, 2763* and *3453*). Illustration by Doris Franke.

Deciduous shrub, 1.5–2.5 m tall; stems glabrous except for strigillose pubescence at the nodes; internodes short, 15–80 mm long (Fig. 5A). Leaves simple, opposite, petiole 8–17 mm long with appressed pubescence; blades elliptic, 50–95 × 15–30 mm, base obtuse, apex acute, margins minutely serrulate, hairs appressed on both sides, longitudinal nerves 3(5) from the base, somewhat prominent adaxially, strongly so abaxially and with simple appressed pubescence (Fig. 5B, C). Inflorescence terminal, 1–4 flowered, the flowers 5-merous; pedicels 2–3.5 mm long with strigillose pubescence (Fig. 5D). Bracts 1 pair per flower, at the base of the hypanthium, pubescent outside, 5–8 × 2–5 mm, caducous. Hypanthium broadly campanulate, 8.5–10 mm long, 7–8.5 mm in diameter in fully opened flowers, with scattered sessile appendages bearing simple penicillate hairs, arranged radially and increasing in size from bottom to top. Intersepalar appendages present, elliptic, c. 2 mm long, with short appressed pubescence and a tuft of long hairs at the apex, caducous. Calyx-lobes uniformly pubescent outside, glabrous inside, asymmetrically obovate and notched at apex, 10–15 × 5–8 mm, contorted in aestivation and completely concealing the floral buds, caducous. Petals 26–30 × 30–40 mm, mauve, broadly obovate, glabrous except for the ciliate margin. Stamens 10, dimorphic in size; antesepalous stamens 5, filament 8–12 mm long, pedoconnective 15–18 mm long, curved, ventral appendage bipartite 4–6 mm long, anther falcate, 10–14 mm long; antepetalous stamens 5, filament 7–10 mm long, pedoconnective 3.5–4.5 mm long, ventral appendage bipartite, ca. 1 mm long, anther falcate, 8–13 mm long (Fig. 5E). Ovary with a crown of persistent bristles, style 25–30 mm long, red, glabrous; stigma simple. Fruit and seeds not seen.

#### Additional specimens examined.

EQUATORIAL GUINEA. Río Muni: Monte Alén National Park, Monte Alén 2 rock slab, 1°40'N, 10°17'E, 1125 m, 12 Feb 2001 (ster.), *Parmentier & Esono 1530* (BRLU0000197!); Monte Alén National Park, Engong rock slab, 5 km west of Engong village, 1°37'N, 10°18'E, 1100 m, 10 May 2002, (fl. buds), *Parmentier & Esono 2721* (BRLU0000195!); Monte Alén National Park, Monte Alén 2 rock slab, 1°40'N, 10°17'E, 1110 m, 27 May 2002 (fl.), *Parmentier & Esono 3453* (BRLU0000196!).

#### Etymology.

The species is named *alenensis* after Monte Alén range and national park in Equatorial Guinea, where it is apparently endemic.

#### Distribution and habitat.

*Nothodissotisalenensis* is endemic to Monte Alén National Park in Equatorial Guinea (Rio Muni), where it occurs in low shrubby vegetation on rocky outcrops (“manteau arbustif”) at ± 1100 m a.s.l. (Fig. 4).

#### Phenology.

Flowering in May.

#### Conservation status.

Vulnerable [VU D2]. *Nothodissotisalenensis* is endemic to Monte Alén National Park in Equatorial Guinea, where it has been collected four times and is known from two rock outcrops, representing two subpopulations. Its EOO cannot be calculated (since only two sites are known) while its AOO is estimated to be 8 km^2^, within the limit for Critically Endangered status under criterion B2. The species occurs in a remote area within a national park, and there is no evidence of an immediate threat or of a population decline. However, its extremely limited range makes it vulnerable to any threat that might arise in the future, e.g. climatic change or introduction of invasive species; it is therefore assessed as Vulnerable according to criterion D2.

##### Key to African Melastomateae genera

**Table:** 

1	Calyx either truncate or with lobes not contorted, leaving the corolla exposed in bud; trees or shrubs with 4-merous flowers; seeds often provided with dorsal hyaline papillae	*** Dichaetanthera ***
–	Calyx-lobes always developed, contorted and concealing the corolla in young bud stage; herbs, or if shrubs then flowers always 5-merous; seeds tuberculate, smooth, ridged or foveolate, without hyaline papillae	**2**
2	Flowers involucrate, solitary or in heads; calyx-lobes persistent; intersepalar appendages absent; fruits capsular or baccate	**3**
–	Flowers not involucrate (except in *Dissotisspeciosa*), solitary, glomerulate, panicled or racemose; calyx-lobes caducous or persistent; intersepalar appendages present; fruits capsular or irregularly dehiscent, never baccate	**5**
3	Shrub with 1-flowered inflorescences; stamens isomorphic, with two ventral and one dorsal appendage, no distinct pedoconnective and erect anthers	*** Cailliella ***
–	Herbs, or if shrubs then flowers several per inflorescence; stamens not as above, with two ventral appendages only, and pedoconnective usually well-developed	**4**
4	Fruit fleshy, baccate; stamens isomorphic (except *T.cornifolium*), with erect anthers; hypanthium with hairs often arranged in rings (but sometimes glabrous or hairy all over)	*** Tristemma ***
–	Fruit dry, capsular; stamens heteromorphic (except *M.porteresii*), with anthers patent or curved; hypanthium glabrous or with hairs not arranged in rings	*** Melastomastrum ***
5	Stems, leaves, and inflorescences with stellate hairs, sometimes with simple hairs present as well; leaves sessile, amplexicaul	*** Argyrella ***
–	Stems, leaves and inflorescences with simple or dendritic hairs, or sometimes glabrescent; leaves usually petiolate	**6**
6	Shrubs	**7**
–	Herbs, sometimes ± woody at base	**11**
7	Calyx-lobes notched at apex, asymmetrical, caducous; leaves deciduous	*** Nothodissotis ***
–	Calyx-lobes entire, ± symmetrical, caducous or persistent (always persistent if leaves deciduous)	**8**
8	Intersepalar appendages absent or reduced to a bristle; calyx-lobes persistent; hairs on stems and leaves ± bulbous at base; leaves deciduous or not	**9**
–	Intersepalar appendages well-developed; calyx-lobes caducous (sometimes tardily so); hairs on stems and leaves not bulbous at base; leaves never deciduous	**10**
9	Inflorescence a 1−3(−7) flowered cyme; intersepalar appendages present; stamens isomorphic; leaves small, 1.5−2.5 × 1−1.5 cm	*** Dionychastrum ***
–	Inflorescence a many-flowered panicle; intersepalar appendages absent; stamens usually dimorphic; leaves much larger	*** Dissotidendron ***
10	Anthers isomorphic, opening by an extrorse pore; leaves distinctly bicoloured, dark green above and yellowish-green beneath	*** Pseudosbeckia ***
–	Anthers usually dimorphic, opening by an introrse pore; leaves not as above	***Dissotis* (*D.* sects. *Macrocarpae*, *Squamulosae*, *Sessilifoliae*)**
11	Calyx-tube accrescent in fruit, developing a long neck with longitudinal ribs; intersepalar appendages absent; stamens dimorphic	*** Dupineta ***
–	Calyx tube not accrescent (except *Dissotistubulosa*) and lacking longitudinal ribs; intersepalar appendages usually present; stamens dimorphic or isomorphic	**12**
12	Seeds longitudinally ridged or foveolate, with a (sometimes very short) basal aril; stems creeping at base; staminal appendages bilobed or bipartite in outer stamens; hypanthium with stalked stellate emergences (except *H.decumbens* with simple hairs)	*** Heterotis ***
–	Seeds tuberculate or smooth, not arillate; stems usually erect, or if creeping (*Guyonia*), then staminal appendages entire in outer stamens; hypanthium usually glabrous or with simple hairs (in *Antherotoma* sometimes with stellate emergences)	**13**
13	Seeds with parallel rows of tubercles; staminal appendages bilobed or bipartite; flowers 4- or 5-merous; calyx-lobes persistent or caducous; hypanthium with simple eglandular hairs or stellate emergences, rarely glabrous (*Chaetolepisgentianoides*)	***Dissotis* and allies (*Antherotoma*, *C.gentianoides*, D.sect.Dissotis, *D.congolensis*, *D.tubulosa*, African “*Osbeckia*”)**
–	Seeds smooth, or with tubercles not arranged in parallel rows; staminal appendages entire in the outer whorl at least; flowers usually 5-merous (4-merous in *Guyoniarupicola*) calyx-lobes always persistent; hypanthium glabrous or with glandular hairs, rarely (*Guyoniapygmaea*) with simple eglandular hairs	**14**
14	Stems thick and fleshy, winged, erect; hypanthium with prominent longitudinal nerves; inflorescence an elongate cyme (raceme) with two well-developed bracts under each flower; plant almost glabrous except the ciliate leaves and calyx	*** Anaheterotis ***
–	Stems slender, usually not winged, creeping or more rarely erect; hypanthium with nerves hardly distinct; inflorescence not as above, usually with very small bracts, often 1-flowered; plant glabrous to densely hairy	*** Guyonia ***

## Supplementary Material

XML Treatment for Nothodissotis

XML Treatment for Nothodissotis barteri

XML Treatment for Nothodissotis alenensis
